# Two-component systems in *Streptomyces*: key regulators of antibiotic complex pathways

**DOI:** 10.1186/1475-2859-12-127

**Published:** 2013-12-19

**Authors:** Héctor Rodríguez, Sergio Rico, Margarita Díaz, Ramón I Santamaría

**Affiliations:** 1 Instituto de Biología Funcional y Genómica (IBFG)/Departamento de Microbiología y Genética, Consejo Superior de Investigaciones Científicas (CSIC)/Universidad de Salamanca, C/ Zacarías González, n° 2, 37007 Salamanca, Spain

**Keywords:** *Streptomyces*, Two-component systems, Antibiotic production, Secondary metabolism

## Abstract

*Streptomyces,* the main antibiotic-producing bacteria, responds to changing environmental conditions through a complex sensing mechanism and two-component systems (TCSs) play a crucial role in this extraordinary “sensing” device.

Moreover, TCSs are involved in the biosynthetic control of a wide range of secondary metabolites, among them commercial antibiotics. Increased knowledge about TCSs can be a powerful asset in the manipulation of bacteria through genetic engineering with a view to obtaining higher efficiencies in secondary metabolite production. In this review we summarise the available information about *Streptomyces* TCSs, focusing specifically on their connections to antibiotic production.

## Introduction

Microorganisms included in the genus *Streptomyces* are Gram-positive bacteria that inhabit soil niches, thus facing ever changing environmental conditions and nutrient scarcity [1]. Along evolution, this challenging environment has pushed the genus *Streptomyces* towards complex adaptive responses. Among them, two-component systems (TCSs) are the most important transduction signal mechanism in bacteria, allowing the translation of these rapid environmental or nutritional changes into a regulatory readout [2,3]. Typically, TCSs comprise a membrane-bound histidine kinase (HK), which senses specific environmental stimuli, and a cognate regulator (RR), which mediates the cellular response, mainly through the transcriptional regulation of target genes [4].

Bacteria belonging to the genus *Streptomyces* harbour a high number of TCSs in comparison with other bacterial genera, probably due to the changing environment that these organisms must inhabit. As an example, the genome sequence of the model *S. coelicolor* has revealed an unprecedented proportion of regulatory genes (approximately 12.3% of the total ORFs); [5,6]. Table 1 summarizes the number of TCSs in all *Streptomyces* species sequenced at the time of writing (P2CS: http://www.p2cs.org).

**Table 1:** **Number of histidine kinases, response regulators and mis-Predicted TCS proteins present in the *Streptomyces *** species sequenced to date*

**Organism**	**Histidine**	**Response**	**Mis-Predicted**
	**Kinase (HK)**	**Regulator (RR)**	TCS protein**
*Streptomyces bingchenggensis* BCW-1	125	117	7
*Streptomyces scabeiei* 87.22	108	95	2
*Streptomyces violaceusniger* Tu 4113	106	99	1
*Streptomyces coelicolor* A3(2)	100	87	1
*Streptomyces avermitilis* MA-4680	91	72	2
*Streptomyces griseus* NBRC 13350	83	80	0
*Streptomyces* sp*.* Sirex AA-E	76	73	2
*Streptomyces flavogriseus* ATCC33331	74	64	1
*Streptomyces cattleya* NRRL 8057	63	59	0
*Streptomyces hygroscopicus* 5008	61	75	5

* Taken from the P2CS database (http://www.p2cs.org/) [72,73].

**mis-Predicted TCS proteins indicates all the TCS proteins missed in the original genome annotation and later identified from DNA ORFs prediction [73].

The competitiveness of these bacteria for resources is also increased due to the production of a large number of secondary metabolites with different activities such as fungicides, cytostatics, modulators of the immune response, and plant growth effectors [1,7]. Henceforth, all these compounds will be grouped under the name “antibiotics” in order to simplify the review. Almost half of all known antibiotics are produced by actinomycetes, mostly *Streptomyces*[1,8,9], including two-thirds of the clinically useful antibiotics [10]. For example, *S. coelicolor* produces three chromosomally encoded antibacterial compounds: actinorhodin (ACT), undecylprodiginine (RED) and calcium dependent antibiotic (CDA). More recently, a yellow pigment (yCPK) associated with a type I polyketide synthase cluster (*cpk*) has also been described [11,12]. However, its genome contains the information necessary to potentially encode more than twenty secondary metabolites, most of them as yet undetected. Many silenced pathways have been observed in all the *Streptomyces* genomes sequenced to date, indicating the high biosynthetic potential of these organisms [13,14].

Antibiotic production responds to stress situations (mainly nutrient starvation) in coordination with primary metabolic responses [15]. Accordingly, *Streptomyces* needs to finely modulate such production, depending mostly on the primary metabolic flux and availability of both nutrients and precursors for these antibiotics [16].

**Figure 1:**
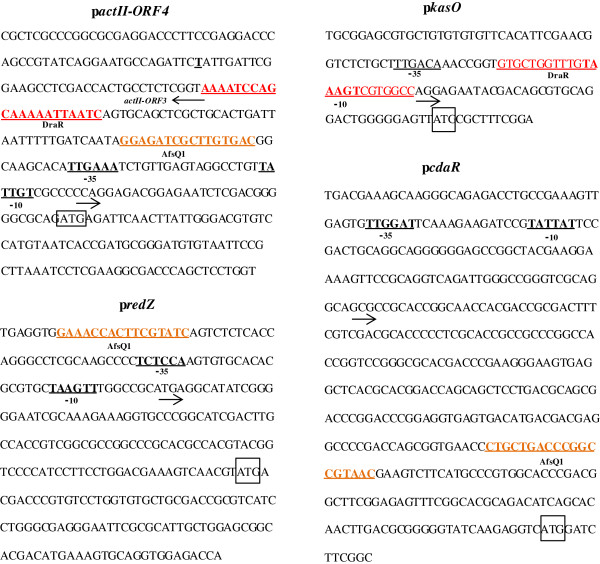
**Location of the binding sequences for the *S. coelicolor *response regulators AfsQ1, DraR in the promoter regions of antibiotic cluster-situated regulators: *actII-ORF4*, *redZ*, *kasO*, and *cdaR***[23,24]. Transcription starting points are indicated with an arrow. -10 and −35 sites and translation start codon are also shown.

Such a complex network of antibiotic regulation is controlled at two main levels. At the lower level, the cluster-situated regulators (CSRs), located within the antibiotic biosynthetic clusters, can modulate the antibiotic biosynthetic genes of the cluster in which they are included, and according to recent data they can also regulate the expression of genes located distant from them [17]. So far, in *S. coelicolor* five CSRs have been elucidated: ActII-ORF4 [18], RedD/RedZ [19,20], KasO (also designated CpkO) [21] and CdaR [22], which are responsible for the biosynthesis of ACT, RED, yCPK and CDA respectively. At the upper level, pleiotropic regulators have been shown to control the production of more than one antibiotic. In *Streptomyces*, the most abundant pleiotropic regulators are the TCSs, a significant fraction of which regulates antibiotic production and morphological differentiation. (Orphan regulators such as RedZ with a role in antibiotic production have also been described but we will just focus here in “traditional TCS” with a cognate histidine kinase). TCSs regulators can act by direct binding to CSRs promoters or can act indirectly, through other regulatory pathways. Only a few binding sequences of *S.coelicolor* TCSs regulators to CSRs promoters have been described to date. These binding motifs are shown in Figure 1.

Broadening our knowledge of the involvement of TCSs in the regulation of antibiotic synthesis can contribute to the rational design of new hyper-producer host strains through genetic manipulation of these complex systems. Moreover, strategies involving TCSs can be used to unveil new antimicrobial molecules that are not produced under laboratory conditions. Some excellent broadly-based reviews regarding general antibiotic regulation in *Streptomyces*[15,16,25-28] and describing TCSs in *Streptomyces*[29] have been published. Here, we summarise current knowledge regarding the involvement of TCS in antibiotic biosynthesis in the model streptomycete *S. coelicolor*, describing how each TCS affects antibiotic biosynthesis and providing some examples of their present applications and their possibilities for the future improvement of antibiotic production and discovery.

**Figure 2:**
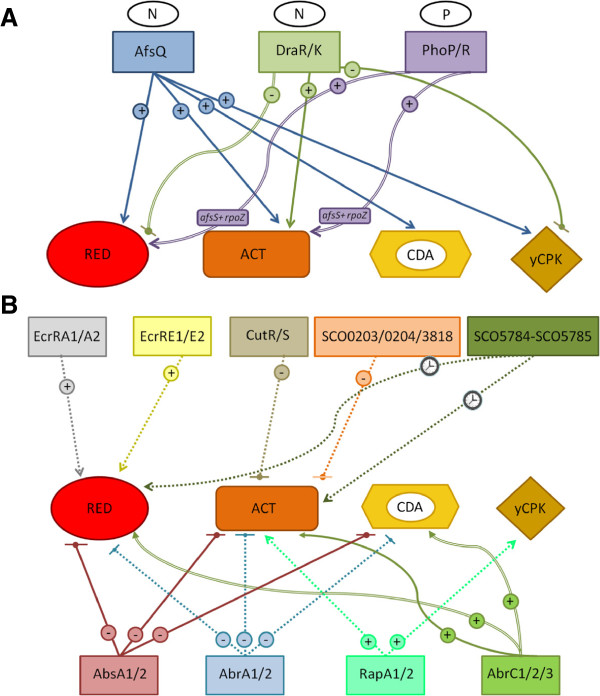
**Schematic overview of the regulation of antibiotic production by the TCSs described in this review. A)** TCSs with a known signal (Nitrogen, N, or phosphate, P). **B)** TCSs with an unknown signal. (+) Indicates positive regulation; (−) indicates negative regulation. Straight arrows indicate that the regulation is exerted through the CSRs. A double-lined arrow indicates that regulation is exerted indirectly, and not through CSRs. The intermediate targets of the indirect regulation are shown, in a box within the arrows, when known. A discontinuous line indicates that the regulation mechanism has not yet been studied. The clock indicates timing control in antibiotic biosynthesis.

To date, the activating signals of most TCSs remain unknown. In light of the available knowledge about the signal triggering the system, we shall divide the review between TCSs with known signals and TCSs whose signals have not been studied and/or that remain unknown. TCSs with unknown signals will in turn be divided between TCSs that regulate the production of a single antibiotic, TCSs that regulate the production of more than one antibiotic, and the regulators responsible for controlling antibiotic production and morphological differentiation. In order to facilitate our understanding of this complex network of interactions, Figure 2 shows updated information regarding which of the *S. coelicolor* TCSs plays a role in the production of each antibiotic and how these regulatory processes work.

## Review

### TCSs with known activating signals

#### Coupling nitrogen availability and antibiotic production: the AfsQ1/2 and DraR/K systems

The AfsQ1/Q2 system was initially identified due to the ability of a *S. coelicolor* fragment containing *afsQ1* to confer the capacity to produce pigmented antibiotics when introduced into a plasmid in *S. lividans,* whose antibiotic gene clusters are usually silenced in most culture conditions [30]. Nevertheless, a deletion mutant of the *S. coelicolor afsQ1* and *afsQ2* genes (Δ*afsQ1/Q2*) failed to produce any phenotype when cultivated in rich medium [30], although when grown on defined minimal medium with glutamate as the only carbon source the Δ*afsQ1/Q2* mutant showed a decrease in ACT, RED and CDA, antibiotic production [31], indicating the different roles of this system, depending on the culture medium. The complementation of the *S. coelicolor* double mutant with the regulator (*afsQ1*) did not restore ACT production, pointing to AfsQ2 kinase as the only phosphorus donor of the response regulator. Although the real signal has not been determined experimentally, a nutritional signal -either an intermediate of nitrogen metabolism or the C/N/P ratio- might act as the trigger of the system.

Regarding the target genes of the AfsQ1 regulator, electrophoretic mobility shift assays (EMSAs) and quantitative RT-PCR (qRT-PCR) experiments revealed that AfsQ1 activates antibiotic biosynthesis by interacting directly with the CSR-genes *actII-ORF4*, *cdaR,* and *redZ.* Moreover, different AfsQ1 binding motifs in the *cdaR*, *redZ* and *actII-ORF4* promoter regions have been described using Dnase I footprinting assays (Figure 1) [23,31]. AfsQ1 also activates *sigQ*, a putative sigma factor that, by acting as a negative regulator of antibiotic production, (*sigQ* deletion leads to an increase in antibiotic levels) might play a role as an antagonist for the AfsQ1/Q2 system [23].

AfsQ1-binding sequences have been found within the *cpkA/cpkD* intergenic region and deletion of *afsQ1/Q2* led to a substantial reduction of the yellow pigment yCPK [23]. The sequences have also been located in genes with roles in morphological development and carbon, nitrogen and phosphate metabolism, indicating that AfsQ1/Q2 responds to nitrogen excess not only by regulating antibiotic production but also by coordinating the C/N/P balance in the cell through the regulation of genes involved in nitrogen assimilation and phosphorus and carbon uptake [32].

The role of the DraR/K system in the regulation of antibiotics biosynthesis was elucidated in a screening of the TCS gene deletion library using minimal medium (MM) supplemented with different nitrogen sources [24], suggesting an interconnection between the role of AfsQ1/2 and DraR/K in response to nutritional signals.

Deletion of *draR/K* (and similarly ∆*draR* and ∆*draK* single mutants) resulted in a reduction in ACT levels but led to the overproduction of RED when grown in high nitrogen concentrations (mainly glutamine). An increase in the yellow pigment (yCPK) in Δ*draR/K* was also observed under the same culture conditions, indicating that the TCS might act as a repressor for RED and yCPK biosynthesis under these circumstances. Thus, the DraR/K system was the first TCS identified that acts differentially in antibiotic biosynthesis in *S. coelicolor*: it is an activator of ACT and a repressor of RED and yCPK. Scanning electronic microscopy revealed that this TCS was also related to morphological differentiation [24].

qRT-PCR assays confirmed that the deletion of *draK/R* originates a decrease in the expression of *actII-ORF4,* the CSR of ACT, and an increase in the expression of *kasO*, the CSR of yCPK. EMSAs assays with the DraR regulator revealed the direct interaction of the DraR regulator with the upstream regions of *actII-ORF4* and *kasO* through the binding of an 11 bp consensus motif defined using DNase I footprinting assays (Figure 1). In contrast, the increase in RED production observed in the double mutant is not related to a higher expression in *redD,* the CSR of RED production [24].

Since AfsQ1/Q2, the TCS described above, had a similar pattern of nitrogen-dependent regulation [31], a double mutant Δ*draR/∆afsQ1* was constructed to study the possible coordination between both TCSs in the activation of *actII-ORF4*. The *actII-ORF4* transcript was significantly decreased in the double mutant (Δ*draR/∆afsQ1*) as compared with the single mutant (Δ*draR*), indicating a possible additive effect between the two systems in the regulation of *actII-ORF4* in glutamate-based medium [24].

Similarly to AfsQ1/Q2, the signal that activates the DraR/K system might be a common intermediate generated during nitrogen metabolism or changes in the C/N ratio under the stress of higher concentrations of nitrogen [32]. Further studies addressing the biochemical and biophysical properties of the extracellular sensory domain of DraK have recently shown that conformational changes in this particular domain occur depending on the pH. This change may be involved in signal transduction processes in DraR/DraK TCS [33]. The recent crystallization of the extracellular sensory domain of DraK might provide new clues to unravel the structure and sensing mechanism of DraK kinase [34].

#### Coordinating phosphate availability and antibiotic production: The PhoP/R system

The TCS PhoP/R is the major signal transduction system for phosphate control in *Streptomyces*. Under phosphate limitation conditions, this TCS plays an important role, activating pathways for phosphate scavenging and controlling the transition to the stationary phase and secondary metabolism [35,36]. Its involvement in the control of primary and secondary metabolism in response to phosphate availability was first described in *Streptomyces* as a result of the search for similarities in the *S. lividans* genome with the Pho regulon of *Escherichia coli* and *Bacillus subtilis*[37-40]. Under inorganic phosphate limitation, deletion of the gene regulator *phoP* (Δ*phoP*) in *S. coelicolor* resulted in a lower and delayed production of ACT and RED. However, EMSA assays did not reveal any binding of PhoP to the promoter regions of the CSRs *actII-ORF4* and *redD*, suggesting an indirect regulation of antibiotic production [41].

*In silico* analysis looking for PHO boxes, the target sequences of the PhoP regulator [42,43], detected a putative binding sequence in the upstream region of the *afsS* gene, previously described as an activator of both ACT and RED in *S. coelicolor*[44]. Interestingly, the PhoP-binding sequence determined by footprinting analysis coincided with the binding region previously reported as the binding region of the AfsR regulator that originates competition between both regulators [45]. Luciferase reporter experiments also confirmed that the PhoP regulator not only competes with the AfsR regulator but also acts as a transcriptional repressor of *afsS.* Additional EMSA assays revealed a reciprocal regulation between both regulators due that AfsR is able to bind the *phoRP* promoter [41]. In addition, it has been shown that PhoP binds to the promoter of the polymerase omega factor gene *rpoZ*, required for the biosynthesis of ACT and RED [46]. These data suggest that PhoP indirectly regulates antibiotic production through AfsS and RpoZ. Briefly, PhoR phosphorylates PhoP when phosphate concentrations decrease, and activated PhoP finally produces *afsS* repression and *rpoZ* activation, yielding an overall positive regulatory effect on antibiotic production in *S. coelicolor*[46].

### TCSs with unknown activating signals

#### TCSs regulating the biosynthesis of one antibiotic

##### CutR/S, SCO0203/0204 and SCO3818 regulating ACT biosynthesis

CutR/S was initially described in *S. lividans,* being the first TCS described in Gram-positive bacteria of the genus *Streptomyces*[47]. Gene replacement mutants of *cutR* and *cutS* exhibited an accelerated and increased production of ACT in different media.

The involvement of CutR/S in antibiotic production was also demonstrated in *S. coelicolor.* After the cloning of *cutR* from *S. lividans* in this organism, an important repression of ACT production was observed. Therefore, CutR/S also negatively regulates ACT production in *S. coelicolor,* although no direct binding to *actII-ORF4* has been demonstrated. There is no information regarding the activator signal of the system [48].

SCO0203/0204 TCS was studied after the finding of high similarity between the regulator of the TCS, SCO0204, and an orphan regulator, SCO3818. The hypothesis that both regulators might be regulated by the same histidine kinase encoded by SCO0203 was confirmed using trans-phosphorylation analysis [49]. Regarding their role in antibiotic production, both deletion mutants (Δ*SCO0203,* Δ*SCO3818*) and the double mutant (Δ*SCO0203/0204*) showed an earlier and increased ACT production in certain complex media sufficient in Mg^2+^. In all cases, overproduction could be complemented through the integration of a functional copy of the deleted gene/s. However, the double mutant (Δ*SCO0203/0204*) could be complemented by a functional copy of *SCO0203/0204* or only by a copy of the kinase encoded by *SCO0203*. The fact that functional complementation of the whole system can be achieved by complementing only with the kinase seems to indicate that there is a functional correlation between the regulator SCO3818 and its potential phosphor donor kinase SCO0203 [49]. Since the phenotype was only evident in complex media sufficient in Mg^2+^, it is reasonable to speculate that this bivalent cation might act as a signalling molecule to activate the system, although it has not yet been defined as the actual signal itself.

##### EcrA1/A2 and EcrE1/E2 regulating RED biosynthesis

Microarray analysis revealed two TCSs designated *ecrA1/A2*[50] and *ecrE1/E2*[51] in the vicinity of the *red* locus that are expressed in coordination with the genes of the RED biosynthetic pathway. Single-deletion mutants of both systems, *ecrA1/A2* and *ecrE1/E2*, originated strains with lower RED production than the wild-type strain, while ACT values did not change significantly between the strains tested. In light of this result, both EcrA1/A2 and EcrE1/E2 can be thought to play a role as positive regulators for RED production [50,51]. *ecr* genes are also present in other *Streptomyces* such as *S. flavogriseus* and *S. venezuelae* that do not harbour the RED cluster. Therefore, although a coordinated expression of *ecrA1/A2* and *ecrE1/E2* with RED cluster genes has been described in *S. coelicolor*, these TCSs should have a different regulatory role in other *Streptomyces* with no RED biosynthetic pathway.

#### TCSs controlling the biosynthesis of more than one antibiotic

##### AbsA1/A2 coordinating antibiotic production

AbsA1/A2 was one of the first TCSs to be related to antibiotic production in *S. coelicolor*[52]. A screening of mutants produced by UV mutagenesis revealed four mutants blocked in antibiotic production without being affected in morphological differentiation. All of them were mutants in a putative TCS designated AbsA1/2 [52]. Further studies in the mutations that originated the non-antibiotic-producing phenotype revealed point mutations in the transmitter domain of AbsA1 histidine kinase, locking the regulatory system into a negatively regulating mode [53] caused by the lack of phosphatase activity in AbsA1 kinase [54]. Surprisingly, both disruption and deletion mutants at the *absA1* and/or *absA2* loci originated a precocious hyper production of ACT and RED antibiotics (Pha phenotype), pointing to AbsA1 as the only kinase able to phosphorylate the regulator [52] and to the activated regulator AbsA2-P as a repressor of antibiotic production. Amino acid replacements of D54E, D54A and D54N in AbsA2, which have been described previously as phosphorylation inhibitors, also caused a Pha phenotype in all three mutants [55].

Biochemical studies support the role of AbsA2-P as a repressor of antibiotic production and have demonstrated that the AbsA1 cytoplasmic domain exerts a dual activity and can phosphorylate and dephosphorylate AbsA2. As expected, antibiotic production is dramatically reduced in mutants with enhanced kinase activity and in those with impaired AbsA1 phosphatase activity [54].

The molecular bases for the Pha phenotype were established by AbsA2-P chromatin inmunoprecipitation (ChIP) and revealed that the phosphorylated regulator binds the promoter regions of *actII-ORF4*, *cdaR* and *redZ*, all of them CSRs [56]. *In vivo* binding targets were confirmed *in vitro* with EMSA experiments [56].

To gain further insight into the signal response mechanism of the system, kinase transmembrane topology was studied by using different AbsA1 C-terminal deletion fusions to eGFP that positioned the fluorescent protein between each of the five predicted transmembrane domains. The results matched the *in silico* topological predictions, demonstrating that the AbsA1 kinase has 5 transmembrane domains and a large extracellular C-terminal domain that might be important for response to a hitherto unidentified signal [57].

##### RapA1/2

A screening of TCSs knock-out mutants in *S. coelicolor* allowed the isolation of the Δ*rapA1/A2* strain, which showed a significant reduction in ACT production but no differences in morphological differentiation or growth on R4C solid medium [58].

Semiquantitative RT-PCRs analysis demonstrated that the expression of *actII-ORF4*, the CSR of the ACT gene cluster, and the two ACT biosynthetic genes, *actIII* and *actVA5,* were clearly reduced in the mutant, suggesting that reduced ACT production may be directly or indirectly dependent on the cluster situated activator *actII-ORF4*[58].

Proteomic analysis of the knock-out mutant also revealed a lower production of KasO, the CSR of the cryptic polyketide biosynthetic gene cluster (*cpk*) responsible for yellow pigment (yCPK) production and this result was confirmed by semiquantitative qRT-PCR [58].

#### TCSs controlling pleiotropic processess

##### AbrA1/2 and AbrC1/2/3 coordinating growth phase and antibiotic production

The deletion of several TCSs with sequence similarity with the above-described negative regulator AbsA1/2 system allowed the identification of two TCSs with opposite activities. While the Δ*abrA1/2* strain displayed a conditional (medium-dependent) increase in antibiotic production and differentiation rates, the deletion of the AbrC1/2/3 (Δ*abrC1/2/3*) system originated a conditional decrease in differentiation rates and antibiotic production. Therefore, both are pleiotropic regulators being AbrA1/2 negative and AbrC1/2/3 positive regulators respectively [59].

Owing to the presence of two histidine kinases in the vicinity of a regulator, the AbrC1/2/3 system should be considered an atypical two-component system. Moreover, each gene is separated from the upstream ORF by a DNA sequence long enough to harbour its own promoter. Therefore, each gene might be expressed independently in order to fit the different needs of bacteria, although the signals detected by these kinases have not yet been identified. Interestingly, this system is conserved in all *Streptomyces* species sequenced so far.

Genome-wide ChIP-chip experiments have demonstrated that the AbrC3 protein is able to bind *in vivo* to the promoter of the CSR of the ACT gene cluster *actII-ORF4p,* explaining the downregulation of the *act* cluster observed in the *ΔabrC3* strain (Rico et al. unpublished data). However, no direct binding of the regulator to the RED and CDA CSRs was observed, suggesting an indirect regulation of these pathways.

The signal or signals triggering kinase phosphorylation remain unknown. Since the phenotypes are medium-dependent, a signal that is only present in certain media seems to be necessary for the system to be activated. An alternative explanation might be that in culture media in which no change in phenotype was observed other regulatory systems could be active, perhaps masking AbrC1/2/3 activity.

##### SCO5784/5785: controlling the timing of sporulation and antibiotic production

This TCS was originally studied because it shares a certain degree of homology with the *B. subtilis degS-degU* operon that influences protein secretion and the timing and level of antibiotic production [60,61]. ACT synthesis occurs later in the deficient strain than in the wild-type, while the propagation of multicopy *SCO5785* results in a higher production of ACT at earlier stages relative to the wild-type strain. An equivalent result was observed when RED was measured. It was also seen that sporulation was delayed when the level of the regulator gene decreased, whereas its overproduction caused early sporulation [62]. As expected, transcriptomic analysis revealed an up-regulation of the ACT and RED CSRs *actII-ORF4* and *redZ* respectively in the overproducer strain and a downregulation in the deficient strain. In this case, the TCS seems to respond to environmental inputs, modulating the timing of both antibiotic production as well as sporulation, and controlling the transition from primary to secondary metabolism [62].

#### Engineering *S. coelicolor TCS* to improve antibiotic biosynthesis

The emergence of new antibiotic-resistant strains makes the discovery and improvement of the production of new antibiotics major challenges for microbial biotechnology. New tools must be developed to exploit the “hidden biosynthetic potential” available in all *Streptomyces* genomes sequenced to date [63,64]. Thus, the sequencing of the *S. coelicolor* genome [5] has revealed a large number of previously unknown metabolic gene clusters, potential candidates for the production of as yet undiscovered antibiotics and natural products [13]. Among all the possibilities, metabolic engineering using the available knowledge about *S. coelicolor* TCSs has been used successfully to achieve higher antibiotic production efficiencies and to unveil “cryptic” antibiotics not produced previously under laboratory conditions. Below we briefly summarise some relevant examples.

The information gained about *S. coelicolor* TCSs has been used in other *Streptomyces* strains. Homologies with TCSs previously described in *S. coelicolor* have been found in many other *Streptomyces* species and could be used as targets to improve antibiotic production. As an example, DraR/K homologues have been found in six *Streptomyces* strains. The involvement of this system in the biosynthesis of other antibiotics was demonstrated using an *S. avermitilis* Δ*draR/K* strain. Antibiotic production profiles changed dramatically in the deletion mutant as compared to the wild-type strain. Thus, it was observed that DraR-K homologues could be useful targets for the metabolic engineering of *Streptomyces* species [24]. Reciprocally, information obtained in other *Streptomyces* strains might be used in *S. coelicolor* in order to improve antibiotic production.

An alternative strategy that applies the regulatory properties of TCSs is the use of TCS-manipulated strains of *S.coelicolor* as heterologous hosts in order to hyperproduce different antibiotics or natural products. These strains may be either deletion mutant strains or strains in which a kinase or regulator has been overexpressed, resulting in antibiotic overproduction. Recently, at our laboratory the production of the antitumor drug oviedomycin has been optimized using this kind of approach. This molecule, isolated from *S. antibioticus*, shows *in vitro* antitumor activity and induces apoptosis in cancer cell lines [65,66]. In these experiments the Δ*abrA1/A2* strain [59] showed a significant increase in the heterologous production of oviedomycin as compared with the wild-type strain (Santamaria *et al*., unpublished results). Accordingly, the Δ*abrA1/A2* deletion mutant strain might be a good candidate for the heterologous production of natural products. This kind of approach can also be used for the combinatorial biosynthesis of antibiotics. Combinatorial biosynthesis manipulates the genes that encode enzymes in biosynthetic pathways rationally in order to redesign antibiotic structures to create new activities and overcome bacterial resistance to existing antibiotics [67].

A metabolic engineering approach can also be used to unveil cryptic pathways for antibiotic biosynthesis. As mentioned above, some of the first TCSs discovered in *S. coelicolor* were revealed using a similar approach. AfsQ was initially identified due to its capacity to induce antibiotic production when introduced into the non-antibiotic producer *S. lividans*[30]. Similar strategies using the overexpression of regulators have been employed successfully in *S. coelicolor*[68] and other *Streptomyces* species [69] to discover “hidden” antibiotic synthetic pathways. The expression of wild-type or mutated pleiotropic regulators can be used to create modified streptomycetes in which cryptic biosynthetic genes clusters have been activated. The overexpression of kinases has also been used successfully in *S. coelicolor* to modify levels of antibiotic production. *absA1* alleles previously shown to be antibiotic enhancers in *S. coelicolor* (see TCS AbsA1/2) were integrated into heterologous *Streptomyces* to alter its secondary metabolism [70]. New antimicrobial activity was induced in ten streptomycetes and, also as a result of AbsA1 activation, pulvomycin (a broad-spectrum antibiotic) was isolated using this method for the first time in *S. flavopersicus*[70]. Regarding the mechanism underlying the induction of new antimicrobial activities, as has been reported AbsA2 is a negative regulator of antibiotic production that requires phosphorylation to exert its repression [56]. *absA1* alleles could counteract the effects of endogenous AbsA1 protein, dephosphorylating the AbsA2 regulator and therefore allowing the overexpression of totally or partially repressed pathways in *Streptomyces* species in which AbsA1/2 pleiotropic regulation occurs. Thus, the expression of wild-type or mutated pleiotropic regulators can be used as a screening method to search for new antibiotics and induce silent biosynthetic pathways in *Streptomyces*.

**Figure 3:**
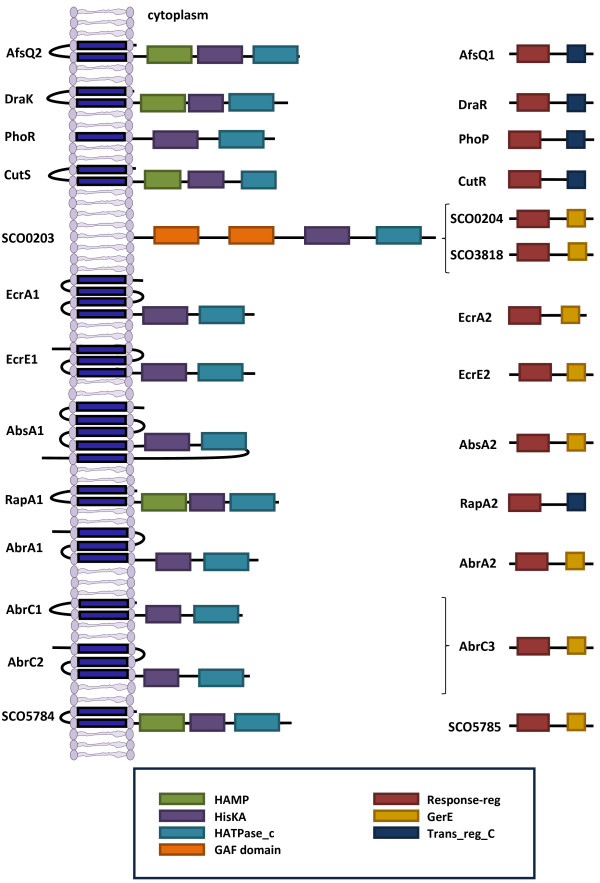
**Schematic representation of the topology of the histidine kinases described and their cognate response regulators in *S. coelicolor*.** The putative transmembrane helices were predicted by TopPred-II [74,75]. Only candidates with a score above 1.0 were considered. The conserved domains and their location are indicated as predicted by PFAM [76].

Another important strategy to decrypt silent pathways in the future might be the use of the proper signals to trigger the TCS activity. As mentioned in previous sections, to date the nature of most of the signals activating *S. coelicolor* TCSs remains elusive. Their discovery and use in different *Streptomyces* might offer an important way forward in the discovery of new metabolites with antibiotic properties [71]. In some cases, topology studies (as we report above in the section addressing AbsA1/A2 TCS) can offer relevant information regarding the position of kinases in the membrane and therefore give up some clues about their signal reception. *In silico* topology predictions of all the kinases described in this review and the conserved domains of both kinases and regulators are shown in Figure 3.

## Conclusions

As discussed above, in recent decades important advances have been made in deciphering the role of TCSs in the regulation of *S. coelicolor* antibiotic production and some examples of applied knowledge have been described briefly. The emergence of an increasing number of antibiotic-resistant strains has made antibiotic research one of the main priorities in biomedical investigations. Here we have shown how antibiotic production can be improved and how the discovery of new antibiotics can be achieved using on-going research into TCSs. More work needs to be done to study new TCSs in *Streptomyces*, the connexions between them, and at the same time the whole regulatory system of these interesting microorganisms.

## Competing interests

The authors declare that they have no competing interests.

## Authors’ contributions

All authors defined the topic of the review and wrote, read and approved the manuscript.
